# Type IV collagen stimulates pancreatic cancer cell proliferation, migration, and inhibits apoptosis through an autocrine loop

**DOI:** 10.1186/1471-2407-13-154

**Published:** 2013-03-26

**Authors:** Daniel Öhlund, Oskar Franklin, Erik Lundberg, Christina Lundin, Malin Sund

**Affiliations:** 1 Department of Surgical and Perioperative Sciences, Umeå University, SE-901 85, Umeå, Sweden

**Keywords:** Type IV collagen, Pancreatic cancer, Basement membrane, Integrin receptors, Autocrine loop

## Abstract

**Background:**

Pancreatic cancer shows a highly aggressive and infiltrative growth pattern and is characterized by an abundant tumor stroma known to interact with the cancer cells, and to influence tumor growth and drug resistance. Cancer cells actively take part in the production of extracellular matrix proteins, which then become deposited into the tumor stroma. Type IV collagen, an important component of the basement membrane, is highly expressed by pancreatic cancer cells both *in vivo* and *in vitro*. In this study, the cellular effects of type IV collagen produced by the cancer cells were characterized.

**Methods:**

The expression of type IV collagen and its integrin receptors were examined *in vivo* in human pancreatic cancer tissue. The cellular effects of type IV collagen were studied in pancreatic cancer cell lines by reducing type IV collagen expression through RNA interference and by functional receptor blocking of integrins and their binding-sites on the type IV collagen molecule.

**Results:**

We show that type IV collagen is expressed close to the cancer cells *in vivo*, forming basement membrane like structures on the cancer cell surface that colocalize with the integrin receptors. Furthermore, the interaction between type IV collagen produced by the cancer cell, and integrins on the surface of the cancer cells, are important for continuous cancer cell growth, maintenance of a migratory phenotype, and for avoiding apoptosis.

**Conclusion:**

We show that type IV collagen provides essential cell survival signals to the pancreatic cancer cells through an autocrine loop.

## Background

Pancreatic cancer is a disease with extremely poor prognosis. The five-year survival rate has only improved marginally over the past decades, and remains at less than 5% [1,2]. Consequently, pancreatic cancer has the lowest long-term survival rate among the common malignancies. The disease typically displays an aggressive, rapid and infiltrative growth pattern, with metastases often already seeded at the time of diagnosis.

Pancreatic cancer is characterized by an abundant fibrotic tumor stroma, referred to as the desmoplastic reaction, which surrounds and infiltrates clusters of cancer cells. Approximately 80% of the tumor mass is made up by the tumor stroma, which is composed of connective tissue, predominantly type I and type III collagen [3], but also a variety of other extracellular matrix (ECM) proteins, blood vessels, inflammatory cells, and activated pancreatic stellate cells (PSC).

In most situations the PSC is a quiescent fat storing cell, but after activation morphological changes occur and it becomes a highly active matrix-producing myofibroblast-like cell, expressing large amounts of type I collagen and fibronectin [4]. Pancreatic cancer cells have been shown to activate the PSC by paracrine mechanisms involving transforming growth factor-β (TGF-β) [5]. The activated PSC is the main producer of the stromal ECM, thus, creating a tumor-supportive microenvironment, resulting in increased tumor growth [5].

The desmoplastic reaction is also believed to be important for the pronounced drug resistance seen in pancreatic cancer. Pancreatic cancer cells attached to type I collagen display increased survival when treated with 5-FU [6], and interactions with other ECM molecules, such as type IV collagen, fibronectin, or laminin, also result in decreased cytotoxicity of anticancer drugs [7]. Furthermore, in a mouse model of pancreatic cancer, inhibition of the Hedgehog signaling pathway led to a depletion of the desmoplastic reaction, and subsequently a better response to chemotherapy [8].

Type IV collagen is upregulated in the tumor stroma and found in discontinuous basement membranes (BM) [9,10]. We have previously shown that type IV collagen is highly expressed in close proximity to the pancreatic cancer cells *in vivo*[11]. Type IV collagen is the main component of the BM and provides a scaffold for assembly and mechanical stability, but is also important in cell adhesion, migration, survival, proliferation, and differentiation [12]. Not only is the activated PSC involved in the production of the stromal ECM, but the cancer cells also produce stromal components both *in vivo* and *in vitro*. It has been shown that many pancreatic cancer cell lines and xenografted human pancreatic tumors express ECM proteins both at the RNA and protein level, and most interestingly, type IV collagen synthesis exceeds that of all other ECM proteins examined, including that of type I collagen [13].

Integrin receptors are transmembrane glycoproteins that are the major cell surface receptors for the ECM, consisting of one α and one β subunit. Both α_1_β_1_ and α_2_β_1_ bind type IV and type I collagens, though α_1_β_1_ has a higher affinity for type IV collagen and α_2_β_1_ for type I collagen [12]. Integrin receptors known to bind type IV collagen (α_2_β_1_ and α_1_β_1_) are highly expressed by pancreatic cancer cells [14]. Furthermore, they are also expressed over the entire cancer cell, not only at the basolateral side as in normal pancreatic tissue [15]. Inhibition of integrin β_1_ has been shown to suppress invasion of pancreatic cancer cells *in vitro*[16]. The integrins have multiple binding-sites on the type IV collagen molecule, but the major binding-site for both α_1_β_1_ and α_2_β_1_ integrins has been identified within the collagenous domain of type IV collagen and is referred to as the CB3-region. By using antibodies to block the CB3 region, 80% of cell adhesion to type IV collagen was inhibited on a variety of carcinoma cell lines [17].

In this study we examine the role of cancer cell-derived type IV collagen and its effects on cancer growth, apoptosis, and migration, and provide evidence of type IV collagen as an important autocrine factor for cancer cell proliferation, survival, and migration in pancreatic cancer.

## Methods

### Tissue samples

Tissue samples of pancreatic ductal adenocarcinomas were collected from patients undergoing a curatively aimed pancreatico-duodenectomy at the Department of Surgery at Umeå University Hospital (n = 9). Normal pancreatic tissue samples were collected from patients who underwent surgery in organs close to the pancreas, but where the disease did not involve the pancreas (n = 7). Samples were snap frozen in liquid nitrogen and stored at -80°C until analysis. This study is approved by the Research Ethics Review Board (EPN) of Northern Sweden.

### Cell lines and cell culturing

Two well-characterized human pancreatic adenocarcinoma cell lines were used. HPAC (ATCC, CRL-2119), a human pancreatic adenocarcinoma cell line of ductal origin, is derived from a moderate to well differentiated pancreatic adenocarcinoma [18]. CFPAC-1 (ATCC, CRL- 1918) was derived from a patient with a ductal adenocarcinoma [19]. HPAC was cultured with 1:1 mixture of Dulbecco’s modified Eagle’s and Ham’s F12 medium (DMEM/F12), CFPAC-1 with 90% Iscove’s modified Dulbecco’s medium (IMDM), both supplemented with 10% fetal calf serum (FCS), penicillin (10^5^ IU/L), and streptomycin (100 mg/L) and grown in an incubator at 37°C in an atmosphere with 5% CO_2_.

### Cell and tissue staining

Immunofluorescence stainings were performed on 6 μm frozen sections, and on cells cultured on Falcon™ Culture Slides (BD Biosciences, Erembodegem, Belgium) using methods previously described [20], with the following primary antibodies and dilutions: mouse anti-human integrin α_1_ (MAB1973, 1:40); mouse anti-human integrin α_2_ (MAB1950, 1:40); mouse anti-human integrin β_1_ (MAB1959, 1:500), all from Millipore (Billerica, MA, USA); and rabbit anti-human type IV collagen (MP Biomedicals, LLC, Solon, OH, USA; cat. no. 10760, 1:100); goat anti-human type XVIII collagen (R&D Systems, Minneapolis, MN, USA; AF570, 1:75), goat anti-human perlecan (R&D Systems, Minneapolis, MN, USA; AF2364, 1:75), rat anti-human laminin-γ1 (Millipore, Billerica, MA, USA, Mab1914P, 1:75), rabbit anti-human nidogen (Millipore, Billerica, MA, USA, cat. no. 481978, 1:75), mouse anti-human cytokeratin 18 (DakoCytomation, Glostrup, Denmark, DC10 1:100) and sheep anti-CD31 (R&D Systems, Inc., Minneapolis, MN, USA; AF806, 1:50). Cultured cells were stained with the integrin antibodies mentioned above together with the goat anti-type IV collagen antibody (Chemicon, Billerica, MA, USA; AB769, 1:50). Double staining on tissue for type I and type IV collagen was performed with a rabbit anti-human type I collagen antibody (Cedarlane Laboratories, Burlington, NC, USA; CL50111AP, 1:200) and a mouse anti-α1(IV)NC1 (Wieslab, Malmö, Sweden; MAB1, 1:75) [21]. Secondary antibodies used were: donkey anti-rabbit FITC, donkey anti-mouse TRITC and FITC, donkey anti-goat TRITC (all Jackson ImmunoResearch Laboratories, Inc., West Grove, PA, USA; with dilutions 1:100). Sections were mounted with medium containing DAPI (Vectashield, Vector Laboratories, Inc., Burlingame, CA, USA). Negative control sections were incubated with secondary antibodies only. Hematoxylin and eosin (H&E) stainings were performed according to standard protocols.

### Migration, proliferation, and apoptosis assays

Cell proliferation was measured using CellTiter-Glo^®^ (Promega Corporation, Madison, WI, USA), a luminescence based cell viability assay, and cell apoptosis was assayed with the M30-Apoptosense^®^ ELISA (Peviva AB, Bromma, Sweden). In the apoptosis assay the apoptosis-associated caspase-cleaved cytokeratin 18 is quantitatively determined, and high concentrations of the M30 neo-epitope indicate high apoptotic activity. Both assays were performed according to the manufacturers’ instructions. For both assays, 5–6 × 10^3^ cells were seeded in triplicates on 96-well polystyrene plates, culture area 0.34 cm^2^/well (Nunc, Roskilde, Denmark), with coated or uncoated surfaces. Proliferation and apoptosis was measured after 2–3 days incubation, and the cells were ensured to be in growth phase when assayed (data not shown). For the apoptosis assay, the triplicates were pooled together and run in duplicates. Furthermore, the fraction of cells in S-phase was measured with flow cytometry, and cells were stained and analyzed according to standard techniques previously described [22]. Transfected cells and control cells were solved in PBS and run in triplicates. DNA histograms were evaluated for ploidy (ModFit LT 3.0™, Verity Software House, Topsham, ME, USA) in order to identify the fraction of cells in the S-phase.

For the matrix studies, type IV and type I collagen (BD Biosciences, Two Oak Park, Bedford, MA, USA) and Bovine Serum Albumin (BSA, used as unspecific control protein) were coated to the wells. For the blocking studies, wells were coated with antibodies directed against the NC1, CB3, and 7S domains of the type IV collagen. These antibodies were: mouse-anti-α1(IV)NC1 (Wieslab, Malmö, Sweden, MAB1); mouse-anti-α2(IV)7S (Chemicon International, Inc., Billerica, MA, USA; MAB1910); mouse anti-human collagen type IV antibody (Acris Antibodies GmbH, Herford, Germany; clone CIV22) [23]; and whole mouse IgG (used as control, Jackson ImmunoResearch Laboratories, Inc.). The coated proteins were diluted in 2 mM hydrochloric acid or 10 mM acetic acid, followed by incubation for 2 hours in the wells. The wells were then washed, cells added, and grown under FCS free conditions.

For the integrin blocking studies, cells were grown in FCS supplemented medium for one day, washed and thereafter, the blocking antibody diluted in serum free medium was added. Antibodies known to functionally block the integrin receptors and a control IgG antibody were used (all mentioned above).

Migration was studied in a wound-healing assay, in which cells were grown on a 24-well wound healing assay plate (Cell Biolabs, Inc., San Diego, CA, USA), and standardized wounds (0.9 mm) were generated according to the manufacturer’s instructions. Two or more wells were used for each experiment, and pictures were taken every 10 minutes (Olympus IX81 with Cell-R software) with time-lapse Differential Interference Contrast microscopy (DIC), and the time of wound closure was determined at two different locations for each wound. Cells were grown under FCS supplemented conditions.

### RNA interference

CFPAC-1 cells were transfected with a pool of 3 different target-specific siRNAs designed to knock down the gene expression of the COL4A1 gene, and control the siRNA design to not target any known human gene (Santa Cruz Biotechnology, Inc., CA, USA, sc-43064 and sc-37007). Subsequently, 5 x 10^4^ cells/ml were seeded in 6 well plates (Nunc, Roskilde, Denmark), and transfected according to the manufacturer’s protocol. The down-regulation of type IV collagen synthesis was verified by quantitative real time-PCR. Approximately 2x10^5^ cells from each cell culture were harvested by centrifugation, and the total RNA was extracted using the RNeasy Mini Kit (Qiagen GmbH, Hilden, Germany). Equal amounts (1 μg) of RNA were reverse transcribed into cDNA using the QuantiTect Reverse Transcription Kit (Qiagen). Gene expression was quantified using the Bio-Rad iQ SYBR Green Supermix and the iQ5 iCycler according to the manufacturer’s instructions. The primer set 5^′^-TGCACCACCAACTGCTTAGC-3^′^ and 5^′^-GGCATGGACTGTGGTCATGAG-3^′^ for the glyceraldehyde 3-phosphate dehydrogenase (GAPDH) housekeeping gene was used for normalization, and the primer set 5^′^-CTCTACGTGCAAGGCAATGA-3^′^ and 5^′^-AGAACAGGAAGGGCATTGTG-3^′^, designed using the web-based PCR primer design tool Primer3 (Rozen and Skaletsky, 2000) was used for quantification of the COL4A1 gene. All reactions were run at 95°C for 3 minutes, followed by 40 cycles at 95°C for 10 seconds and at 60°C for 30 seconds.

Additionally, the type IV collagen protein expression was detected in the transfected cells using immunofluorescence staining with the MAB1 antibody. The transfected cells were assayed for proliferation, apoptosis, and migration as described above. For the cell proliferation assay, the experiments were repeated five times with similar results both under FCS free and serum supplemented conditions.

### Statistics

All assays performed on 96-well plates were run in triplets. For the M30-Apoptosense^®^ ELISA, the triplicates were subsequently pooled and the final analysis was performed on duplicates of the pooled samples. Normal distribution was assumed. Unpaired *t*-test was used when comparing two groups and ANOVA with Bonferroni post-hoc test if more than two groups were compared. P < 0.05 was considered significant. Bars in the figures illustrate the standard deviation.

## Results

### Type IV collagen is highly expressed in the stroma of pancreatic cancer when compared to other basement membrane proteins

The expression of type IV collagen and other BM proteins (type XVIII collagen, laminin, nidogen and perlecan) was studied by immunofluorescence in normal pancreas and pancreatic cancer tissue. In normal tissue all these BM proteins have the same expression pattern with expression in the epithelial and vascular BM (Additional file 1: Figure S1). However, in cancer distinctly different expression patterns were observed, with high expression of type IV collagen in vicinity of the cancer cells. Perlecan and nidogen expression was also observed in the stroma, although the staining pattern and intensity was lower than that observed for type IV collagen. For type XVIII collagen and laminin only little expression was observed in the tumor stroma (Additional file 1: Figure S1).

### Type IV collagen is highly expressed in close proximity to the pancreatic cancer cells and colocalizes with integrin α_1_, α_2_, and β_1_ on the cancer cell surface

**Figure 1:**
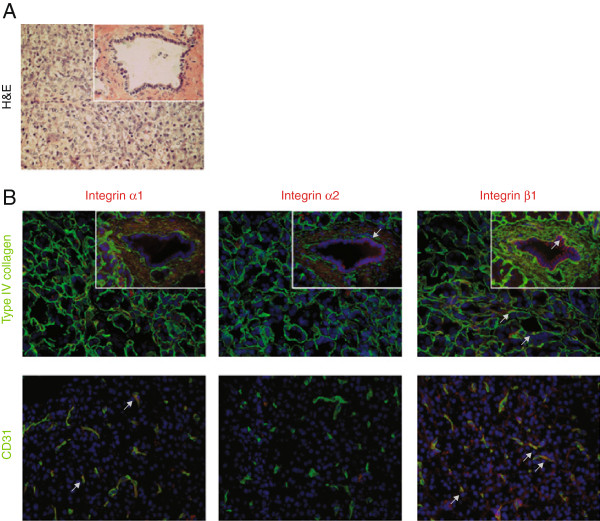
**Expression pattern of integrin receptors and type IV collagen in normal pancreatic tissue. A**. H&E staining of normal exocrine pancreas with acini structures and a duct in the insert. The area shown is representative for all panels in B. **B**. Merged immunofluorescence staining of integrin α_1_, α_2_, and β_1 _(in red), and type IV collagen (green in upper row), or the endothelial marker CD31 (green in lower row). Type IV collagen is present in the vascular-, ductal-, and acini-BMs. Integrin α_1 _is only expressed in endothelial cells and integrin α_2 _only in the ductal epithelium. Integrin β_1 _is found in the ductal-, endothelial-, and at the base of the acini-cells. Arrows indicate examples of colocalization. Magnification x40 in all panels. Cell nuclei are stained by DAPI (in blue).

The expression pattern of type IV collagen in relation to the integrin receptors known to bind type IV collagen (α_1_β_1_ and α_2_β_1_) was examined with immunofluorescence both in normal pancreas and in pancreatic cancer tissue. In normal tissue (Figure 1 and Additional file 2: Figure S2), type IV collagen is present in the vascular-, ductal-, and acini-BMs. Integrin α_1_ is only expressed in the endothelial cells, whereas α_2_ is exclusively expressed at the basolateral surface of the ductal cells. Integrin β_1_ is expressed in the endothelial cells as well as at the basolateral surface of the ductal and acini cells.

**Figure 2:**
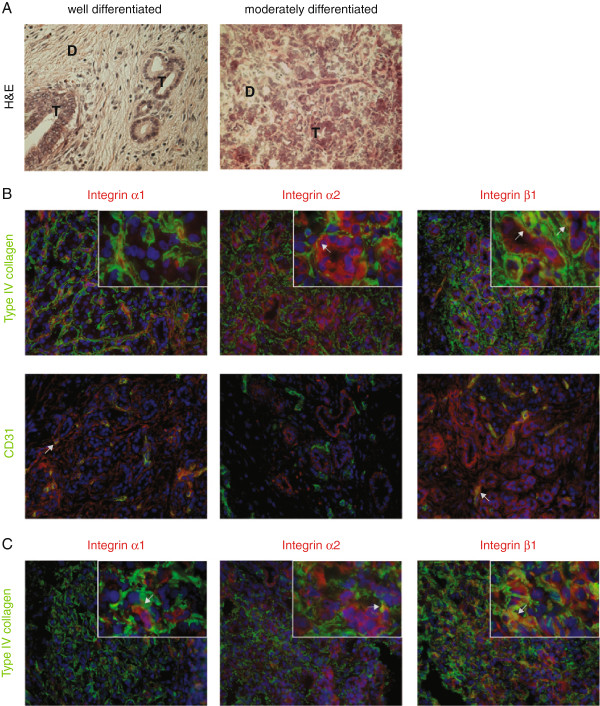
**Expression pattern of integrin receptors and type IV collagen in pancreatic cancer tissue. A**. H&E staining of representative areas of a well (in **B**) and moderately (in **C**) differentiated pancreatic adenocarcinoma. Clusters of tumor cells (T) surrounded by desmoplastic stroma (**D**). B. Merged immunofluorescence double staining of integrin α_1_, α_2_, and β_1_ (in red), and type IV collagen (green in upper row), or the endothelial marker CD31 (green in lower row). Type IV collagen is highly expressed in close vicinity to the cancer cells, forming BM like structures around small clusters of cancer cells. Integrin α_2 _and β_1 _are expressed on cancer cells, and these receptors colocalize with type IV collagen (in yellow). Integrin α_1 _and β_1 _are expressed in the endothelial basement membrane, as indicated by colocalization with CD31. C. In moderately differentiated cancer the same expression pattern as in well differentiated cancer can be seen, but integrin α_1 _is sporadically expressed in cancer cells. Close-up in inserts (magnification x100), all other panels with a magnification of x40. Arrows indicate examples of colocalization. Cell nuclei are stained by DAPI (in blue).

In pancreatic cancer tissue (Figure 2 and Additional file 3: Figure S3), the expression pattern of type IV collagen and the integrin subunits were examined in both well- and moderately differentiated pancreatic cancer. Regardless of the differentiation grade, type IV collagen is highly expressed in close proximity to the cancer cells, forming BM like structures surrounding the cancer cells. Both integrin α_2_ and β_1_ are expressed by the cancer cells, not only basolaterally, but all over the cell surface, and they colocalize with type IV collagen. Integrin β_1_ is also expressed by the endothelial cells, as is integrin α_1_. Moreover, in moderately differentiated cancer cells integrin α_1_ is sporadically expressed by cancer cells and partly colocalized with type IV collagen.

**Figure 3:**
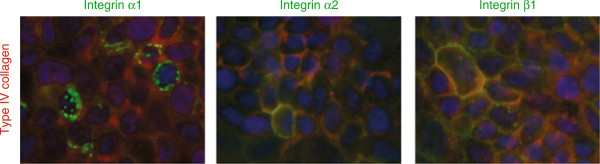
**Expression of integrin receptors in pancreatic cancer cell lines. **Merged images with type IV collagen in red and integrin receptors in green. Type IV collagen is highly expressed by pancreatic cancer cells. Integrin α_1 _is expressed, but is found also intracellularly, not exclusively at the cell surface. Integrin α_2_ and β_1 _are expressed and partly colocalized with type IV collagen (in yellow). Cell nuclei are stained by DAPI (in blue). In the figure HPAC cells are shown, but similar expression pattern was seen with CPFAC-1.

Type IV collagen is also highly expressed at the surface of pancreatic cancer cells *in vitro* (Figure 3 and Additional file 4: Figure S4) and colocalizes with integrin α_2_ and β_1_. Integrin α_1_ is mostly found intracellular and less at the cell surface.

### Both type I and type IV collagens promote growth and migration and inhibit apoptosis, but are expressed in different stromal compartments

**Figure 4:**
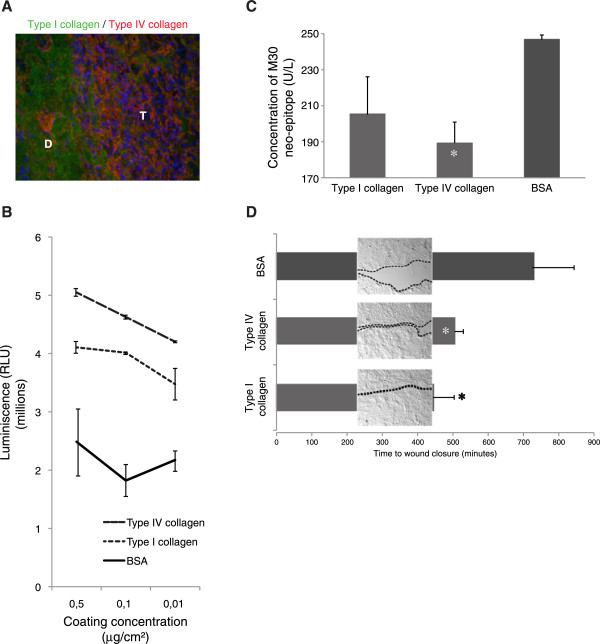
**Expression patterns of type I and type IV collagen and their effect on cell growth. A**. Double staining of type I (in green) and type IV collagen (in red) in a pancreatic adenocarcinoma. Cell nuclei are stained by DAPI (in blue). Type I collagen is predominantly found in the desmoplastic reaction (D) that surrounds and partly infiltrates clusters of tumor cells (T). Type IV collagen is highly expressed in close proximity to the tumor cells with lower expression in the desmoplastic area. Magnification x40. **B**. Growth of pancreatic cancer cells on different matrices after 2 days of incubation. Both type I and type IV collagens promote growth when compared to BSA (used as control protein) in all different coating concentrations (p < 0.05). **C**. Apoptosis measured with the M30-Apoptosense^®^ ELISA after 48 h incubation in serum free conditions with cells grown on different matrices. Type IV collagen inhibits induction of apoptosis compared to a control BSA matrix (* indicates p < 0.05 compared to BSA). **D**. Time for wound-healing closure for cells grown on different matrices (coated with 0.5 μg/cm^2^). * indicates p < 0.05 compared to BSA. Picture-inserts represent the size of the wound at 445 minutes, when the wound on the type I collagen matrix was closed (dotted line indicates cell front). Figures B, C, and D are all based on data from HPAC, but similar results were observed for CFPAC-1.

In pancreatic cancer type I collagen is predominantly expressed in the desmoplastic reaction that surrounds and infiltrates clusters of cancer cells (Figure 4A). However, most of the cancer cells are not in direct contact with type I collagen. Type IV collagen, on the other hand, is highly expressed in close relation to all cancer cells.

Pancreatic cancer cells were grown on both type I or type IV collagen matrices, and both types of collagen promote cell growth when compared to a control protein matrix (Figure 4B). Furthermore, there was a tendency of having reduced induction of apoptosis when the pancreatic cancer cells were grown on collagen matrices (Figure 4C). Migration was measured in a wound-healing assay, in which cells were grown on the different collagens and the time of wound closure was compared to cells grown on a control matrix (Figure 4D). The wounds of cells grown on a collagen matrix closed faster than wounds grown on the control matrix, indicating that cells in contact with type I or type IV collagens develop a more migratory phenotype.

### Type IV collagen is an important autocrine factor regulating growth and migration in pancreas cancer cells

**Figure 5:**
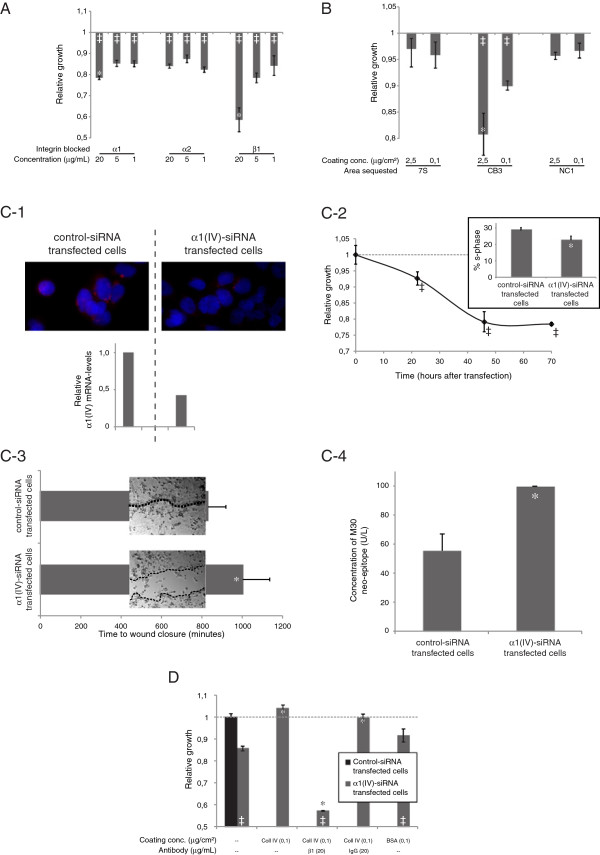
**Cellular effects of type IV collagen. A.** Blocking of integrin α_1_, α_2_, and β_1 _resulted in reduced growth compared to control IgG (‡ indicates p<0.05 compared to index 1.0). Blocking of α_1_ and β_1 _showed dose dependency (*indicates p<0.05 compared to 1 μg/ml). **B. **Effects on cell growth after incubation with antibodies blocking different domains of type IV collagen, presented as relative growth compared to cells incubated with unspecific control IgG antibody (index 1.0). Blocking of the CB3-region reduces growth dose dependently (‡ indicates p<0.05 compared to index 1.0 and *indicates p<0.05 compared to 0.1 μg/cm^2^). **C-1.** Down-regulation of type IV collagen expression at the protein level (α1(IV) in red and DAPI in blue) and the mRNA level in α1(IV) siRNA transfected cells. **C-2.** Reduced cell growth after down-regulation of the α1(IV)-chain. ‡ indicates p<0.05. Growth inhibition was measurable after 20 hours and the effect sustained for 70 hours. Inserts illustrate differences in S-phase, p=0.03. **C-3. **Wound-healing assay for α1(IV)- and control-siRNA transfected cells. Picture-inserts represent the size of the wound at 83s, when the wound in the control cells was closed. *indicates p<0.05 compared to control transfected cells. **C-4. **Increased levels of the apoptotic M30-neoepitope were observed in α1(IV)-siRNA transfected cells (p=0.03). **D. **Under serum free conditions, α1(IV)-siRNA transfected cells show reduced growth. The cell growth is rescued if α1(IV)-siRNA transfected cells are grown on an exogenous type IV collagen matrix. Blocking of integrin β_1 _inhibits the rescue effect, whereas addition of a control IgG antibody does not. If α1(IV)-siRNA transfected cells are grown on an unspecific matrix (BSA), a small positive but non-significant effect on growth was observed. *indicates p<0.05 compared to α1(IV)-siRNA transfected cells grown without any coated matrix and without any blocking antibodies, and ‡ indicates p < 0.05 compared to control-siRNA transfected cells.

In order to further study the interaction between type IV collagen and the integrin receptors observed in pancreatic cancer tissue, a series of *in vitro* experiments were performed in pancreatic cancer cell lines, where different targets in the type IV collagen-integrin axis were manipulated (Figure 5).

By blocking the integrin receptors α_1_, α_2_, and β_1_ a decrease in cell growth was observed (Figure 5A). For integrin α_1_ and β_1_ this growth inhibition was dose dependent.

The integrin receptors predominantly bind to the CB3 region of the type IV collagen molecule, but also to the NC1 domain [12]. By blocking these binding sites with region-specific antibodies, a decrease in cell growth was seen when blocking the CB3 region, but not when blocking the NC1 domain (Figure 5B). The 7S domain is not known to bind to any integrins; hence, blocking this domain did not affect cancer cell growth.

Down-regulation of α1(IV) synthesis was achieved through RNA interference and verified on both the mRNA and protein level (Figure 5C-1). With qRT-PCR, the down-regulation was approximated to a 0.45-fold reduction in α1(V)-transcripts compared with cells transfected with control siRNA. This reduction of endogenous type IV collagen expression in pancreatic cancer cells led to a 20% decrease in cell growth 40 hours after transfection, when compared to cells transfected with control (nonsense) siRNA (Figure 5C-2). The reduction in cell growth was also confirmed by measuring the S-phase (Figure 5C-2 insert). Control cells showed a S-phase of 28.6% compared to 22.6% for the α1(IV)-siRNA transfected cells. In the wound-healing assay, the α1(IV)-siRNA transfected cells showed a less migratory phenotype compared to the control transfected cells (Figure 5C-3 and Additional file 5: Movie S1). In addition, an increased induction of apoptosis was observed in the α1(IV)-siRNA transfected cells when compared with control siRNA transfected cells (Figure 5C-4), indicating that cancer cell-produced type IV collagens can have a protective role against apoptosis.

Furthermore, the reduction of cell growth induced by down-regulation of α1(IV) synthesis was normalized when the cells were grown on a type IV collagen matrix (Figure 5D). This growth rescue effect was not achieved if the cells were grown on a control matrix composed of BSA. Moreover, the rescue once again failed if integrin β_1_ receptors were blocked during the time the α1(IV)-siRNA transfected cells were grown at the type IV collagen matrix (Figure 5D).

Together, these data demonstrate the importance of type IV collagen/integrin receptor interactions and shows that type IV collagen is an important autocrine factor, regulating growth, migration, and apoptosis in pancreas cancer cells.

## Discussion

Previous studies have shown that many integrin receptors are expressed in pancreatic cancer cells [14,16]. Type IV collagen is produced by the cancer cells, and is deposited in direct proximity to the cancer cells in the pancreatic tumor stroma, forming discontinuous BM like structures [9-11], but the functional role of this is unknown. This is not the case for all BM proteins as shown in this study, which indicates that these proteins although expressed in the same structure in normal pancreas tissue, can have different functions in pancreatic cancer. Cancer is a clonal disease and the cancer cells seen in an advanced cancer are all results of many cell generations of active selection, where the cells most fitted for rapid expansion have gained a growth advantage. Protein synthesis is an energy consuming process for all cells, and if type IV collagen synthesis was not of importance for cancer progression, clones with lower collagen production would have emerged and gained advantage in the cancer cell population. Thus, the observed accumulation of BM like structures close to the cancer cells both *in vivo* and *in vitro* must mean that type IV collagen is, somehow, important for the cancer cells. In this study we show that type IV collagen colocalizes with integrins known to bind type IV collagen on the cancer cell surface both *in vivo* and *in vitro*, and that the interaction between the CB3 region of the type IV collagen molecule, and integrin receptors (especially β_1_) on the surface of the cancer cells, is an important factor that stimulates proliferation and migration, and inhibits apoptosis in pancreatic cancer cells. This finding also gives a rationale for the abundant deposition of type IV collagen seen in human pancreatic cancer tissue.

Furthermore, we show that type I collagen also promotes cancer cell proliferation, migration, and regulates apoptosis, in the same extent as type IV collagen, but that these two collagen types are expressed in different compartments of the tumor stroma *in vivo*. The PSC are located in the desmoplastic area and are the main producers of type I collagen in the stroma, but most cancer cells are not in direct contact with type I collagen, whereas type IV collagen is produced by the cancer cells themselves, and forms BM like structures surrounding clusters of cancer cells. Taken together, although it is likely that both type I and type IV collagens are important for pancreatic cancer progression, they might have distinct roles in the pathogenesis of pancreatic cancer.

The integrin-ECM axis in pancreatic cancer is believed to play an important role in regulating growth and migration of pancreatic cancer cells [7]. Mutations in pancreatic cancer cells lead to constitutive activation of TGF-β_1_, which in turn results in the proliferation of pancreatic stellate cells (PSC) [7]. PSCs produce the type I collagen and fibronectin found in the desmoplasia, which in turn mediates cancer cell proliferation and migration through integrin receptors (α_2_β_1_ for type I collagen and α_5_β_1_ for fibronectin)[7]. In addition to this paracrine mechanism, based on our findings, we provide evidence of an autocrine loop of endogenous type IV collagen produced by the pancreatic cancer cell, important in regulating growth, apoptosis, and migration in pancreatic cancer. We hypothesize that pancreatic cancer cells, by producing BM proteins such as type IV collagen, form their own BM-like structures that the cancer cells, in an autocrine manner, can anchor to and consequently, achieve sustained cell growth, avoid apoptosis, and develop a migratory phenotype.

Our data show that reduced production of endogenous type IV collagen, blocking of the CB3-region on the type IV collagen molecule and blocking of the integrin β_1_ receptor, all lead to a pronounced growth inhibition. However, the antibody we used to block the CB3-region has been shown not to affect the binding of integrin α_1_β_1_ to type IV collagen [23]. As expression of integrin α_1_*in vivo* was only occasionally observed on pancreatic cancer cells, and was found mostly intracellular *in vitro,* our results indicate that integrin α_1_ is of less importance for the interaction we have studied, and that integrin β_1_ in combination with another α subunit, forming dimers with affinity to the CB3-region, causes the observed effects.

Attachment to the BM is crucial for the survival and growth of the epithelial cells. Integrin receptors play an important role in anchoring the cells and in providing survival signals, and if the epithelial cell loses contact with the BM, apoptosis is initiated [24,25]. Pancreatic adenocarcinoma is of epithelial origin, and in the transformation process this mechanism must be overrun; otherwise, apoptosis will be induced as soon as the BM is degraded in order to make subsequent tumor growth possible. We believe that pancreatic cancer cells, by producing their own BM, gain proliferative and invasive properties of crucial importance for the pathogenesis of pancreatic cancer. We have studied type IV collagen, one of the most abundant components of all BM and highly expressed by pancreatic cancer cells [9,10,13]. Most certainly other BM proteins are produced and show similar autocrine effects, and we show here that proteins such as nidogen and perlecan are also found in the tumor stroma. Therefore, the tumor produced BM might conceal future therapeutic targets that will be important for the development of new treatment strategies for pancreatic cancer.

## Conclusion

In this study we demonstrate that type IV collagen is produced by the pancreatic cancer cells and forms BM like structures surrounding the cancer cells. Through an autocrine loop, type IV collagen interacts with integrin receptors on the surface on the cancer cells, and stimulates pancreatic cancer cell proliferation, migration, and inhibits apoptosis.

## Consent

Written informed consent was obtained from the patient for publication of this report and any accompanying images.

## Abbreviations

BM: Basement membrane; ECM: Extracellular matrix; PSC: Activated pancreatic stellate cell; TGF-β: Transforming growth factor-β.

## Competing interests

The authors declare no competing interests.

## Authors’ contributions

DÖ and MS designed the study. DÖ, OF, MS, EL and CL performed the experiments. DÖ, OF, MS and EL analyzed the data. DÖ and MS interpreted the data and wrote the paper. All authors read and approved the final manuscript.

## Pre-publication history

The pre-publication history for this paper can be accessed here:

http://www.biomedcentral.com/1471-2407/13/154/prepub

## Supplementary Material

Additional file 1: Figure S1 The expression pattern of various basement membrane (BM) proteins and cytokeratin 18 (CK18) in normal pancreas and pancreatic cancer tissue. No CK 18 expression was observed in normal tissue as expected, whereas strong expression by pancreatic cancer cells can be seen (B,D, F, H and J). The expression patterns of all the BM proteins are the same in normal pancreas tissue (A, C, E, G and I). However, in pancreatic cancer tissue an intensive staining can be observed in the tumor stroma of type IV collagen (B). For nidogen (H) and perlecan (J) staining in tumor stroma can be seen, whereas only weak staining can be observed for type XVIII collagen (D) and the gamma-1 chain of laminin (F). Click here for file

Additional file 2: Figure S2 The expression pattern of integrin receptors and type IV collagen in normal pancreas tissue shown as single channels. The same pictures as in Figure 1B but the different channels are presented individually and not merged. Click here for file

Additional file 3: Figure S3 Expression pattern of integrin receptors and type IV collagen in pancreatic cancer tissue shown as single channels. The same pictures as in the inserts (x100 magnification) of Figure 2B and Figure 2C, but the different channels are presented individually and not merged. A represents well differentiated and B moderately differentiated pancreatic adenocarcinoma. Click here for file

Additional file 4: Figure S4 Expression of integrin receptors in pancreatic cancer cell lines shown as single channels. The same pictures as Figure 3 but the different channels are presented individually and not merged. Click here for file

Additional file 5: Movie S1 Difference in migration for α1- and control-transfected cells. Movie that illustrates the difference in migratory capacity between α1(IV)-siRNA transfected and control-siRNA transfected cells. Click here for file
